# Effects of zilpaterol hydrochloride and virginiamycin on growth performance, carcass characteristics, and visceral organ mass in feedlot lambs

**DOI:** 10.5713/ab.24.0457

**Published:** 2025-08-12

**Authors:** Mario Alejandro Mejía Delgadillo, Karla Hildeliza Leyva Medina, Jaime Noe Sánchez Pérez, Héctor Aarón Lee Rangel, Hugo de Jesús López Inzunza, Roberto Martínez León, Gamaliel Molina Gámez, José Luis Ponce Covarrubias, Horacio Dávila Ramos, Juan Carlos Robles Estrada

**Affiliations:** 1 Facultad de Agronomía, Universidad Autónoma de Sinaloa, Culiacán, México; 2 Facultad de Medicina Veterinaria y Zootecnia, Universidad Autónoma de Sinaloa, Culiacán, México; 3 Facultad de Agronomía y Veterinaria, Universidad Autónoma de San Luis Potosí, San Luis Potosí, México; 4 Escuela Superior de Medicina Veterinaria y Zootecnia No. 3, Universidad Autónoma de Guerrero, Técpan de Galeana, México

**Keywords:** Carcass Characteristics, Growth Performance, Lambs, Virginiamycin, Zilpaterol

## Abstract

**Objective:**

The objective was to determine the effect of dietary zilpaterol hydrochloride and virginiamycin on growth performance, dietary energetics, carcass characteristics, and visceral organ mass in feedlot lambs.

**Methods:**

Thirty-two crossbred lambs Dorper×Katahdin (34.04±4.23 kg), five months old, were used in a 30 d experiment to evaluate the inclusion of zilpaterol and virginiamycin. Treatments were randomly assigned to pens within blocks, with four replicates per treatment. Data were analyzed as a randomized complete block design arranged as 2×2 factorial, with two levels of zilpaterol (0 and 0.20 mg/kg of live weight/d, as hydrochloride form) and two levels of virginiamycin (0 and 22 mg/lamb/d). The diet was based on cracked corn (1.41 Mcal NEg/kg of dry matter and 14.1% of crude protein). Growth performance and dietary energetics variables were recorded. After the feeding trial, lambs were transported to a slaughterhouse for assessment of carcass characteristics, visceral organ mass, and primal cuts.

**Results:**

No interactions were observed between zilpaterol and virginiamycin treatments for most of the evaluated variables, except for the percentage of the empty small intestine. Lambs supplemented with both zilpaterol and virginiamycin showed improvements in live weight, feed efficiency, and energy retention. However, only zilpaterol affected dressing percentage, *longissimus thoracis* muscle, and body fat reduction. The combined supplementation increased final live weight without altering feed intake, thereby enhancing energy availability to promote carcass weight and dressing carcass compared to lambs treated with zilpaterol alone.

**Conclusion:**

Zilpaterol hydrochloride (0.20 mg/kg of live weight/d, equivalent to 7.55 mg/lamb/d) and virginiamycin (22 mg/lamb/d) improved growth and energy retention, but only zilpaterol improved dressing percentage and reduced fat traits of carcass and non-carcass components. The effects of virginiamycin and zilpaterol were found to be cumulative, with better responses in growth and dietary energy components observed in lambs supplemented with both additives.

## INTRODUCTION

Generally, feed additives are included in the diets of farm animals to promote health, enhance growth performance, improve feed efficiency (FE), or alter the characteristics of the feed [1]. Zilpaterol is a β_2_ adrenergic agonist used as a feed additive in feedlot cattle to improve average daily gain (ADG) and FE by up to 26%, as well as to improve carcass characteristics such as hot carcass weight (HCW) by up to 7.5% [2,3]. Zilpaterol’s effects are not permanent, and its benefits in cattle are obtained within 20–30 days of supplementation before slaughter, at a daily dose of approximately 0.15 mg/kg of live weight (LW). Studies in lambs have shown that a daily dose of up to 0.20 mg/kg of LW produces a similar response to the recommended dosage for cattle. This results in ADG and FE improvements of up to 18.3%, along with an increase in HCW of up to 4.8% [4,5]. Since 1995, this additive has been approved for use in beef feedlots in South Africa, in Mexico since 1996, and in the USA since 2006. However, the European Union, Russia, and South Korea have legislation prohibiting the importation of products from animals supplemented with β-adrenergic agonists [6,7].

Virginiamycin is a feed additive derived from the *Streptomyces virginiae* fermentation process, that in cattle supplemented with a dose between 22–28 mg/kg diet of dry matter (DM) has been shown to improve animal growth performance [8]. It primarily improves productivity by affecting rumen microorganism populations and activity, maintaining a stable rumen fermentation pattern when ruminants are fed high-grain diets [9]. Likewise, virginiamycin effectively inhibits the growth of gram-positive bacteria, particularly those of rapid growth that produce lactic acid, such as *Streptococcus bovis* and *Lactobacillus* spp., without impacting lactate-utilizing microorganisms [10]. Furthermore, virginiamycin enhances overall health by decreasing the occurrence of liver abscesses by reducing the population of *Fusobacterium necrophorum* in the rumen [11]. During the 1990s, numerous evaluations were conducted using lambs as a model to study the use of virginiamycin and its effects on nutrient digestion and ruminal fermentation [12,13]. However, in recent years, its impact on growth performance, energy efficiency, and carcass quality in lambs has only recently begun to be evaluated [14–16]. Virginiamycin is currently approved for use in several countries, including the United States and México. However, its use as a growth promoter has been banned in the European Union since 1999 due to concerns regarding antimicrobial resistance, and in South Korea since 2011 [17].

Research on the simultaneous use of β-agonists and antibiotics has primarily focused on the ionophore monensin in cattle [18,19]. The combined supplementation of zilpaterol and monensin in cattle diets has been associated with a reduction in carcass yield grade compared to steers that received no additives, as well as those treated only with zilpaterol. Additionally, it has been suggested that monensin can be withdrawn from the diet during zilpaterol supplementation without negatively affecting productive performance. In feedlot lambs, information on the combined use of zilpaterol and virginiamycin to improve growth performance and carcass characteristics is limited. Since zilpaterol and virginiamycin enhance weight gain and FE through different mechanisms, we hypothesized that their combined use would result in a cumulative effect, leading to improved responses compared to their individual use. Therefore, the objective of this study was to determine the effect of dietary zilpaterol in combination with virginiamycin on growth performance, dietary energetics, carcass characteristics, and visceral organ mass in feedlot lambs.

## MATERIALS AND METHODS

### Facilities and management

This research was conducted in compliance with the animal welfare regulations established by the Mexican government and was approved by the Institutional Committee for the Care and Use of Faculty of Animals of the Veterinary Medicine and Animal Science (CICUA-FMVZ/16-10-2022). Throughout the study, animal welfare was monitored daily by trained personnel to detect signs of distress, discomfort, or adverse effects potentially associated with the feed additives. No adverse clinical signs were observed, and all animals remained healthy throughout the trial, which wasconducted in accordance with the welfare standards defined by the institutional ethics committee. The research was conducted at the experimental unit for small ruminants of the Autonomous University of Sinaloa, located in the city of Culiacán, Sinaloa, Mexico (24.7721° N, 107.3545° W).

Three weeks before the beginning of the experiment, forty intact male Dorper×Katahdin cross lambs were identified and weighed using a hook scale with a precision of 50 g (Torrey, CRS-200). Subsequently, they were adapted to the management conditions, pens, and experimental diet; treated against parasites (Saguaymic plus, Microsules lab), hemoparasites (Imizol, MSD Animal Health), and injected with 1×10^6^ IU of vitamin A (Synt-ADE, Fort Dodge, Animal Health). In the adaptation stage, lambs that showed disease or atypical growth were discarded and were not considered for the experimental stage. From the remaining group, lambs with an average initial LW of 34.04±4.23 kg and aged five months were utilized in a 30-d experiment to evaluate the inclusion in the diet of zilpaterol and virginiamycin on growth performance, dietary energetics, carcass characteristics, and visceral organ mass. The study incorporated pre-slaughter daily supplementation of two levels of zilpaterol hydrochloride (0 and 0.20 mg/kg of LW) and two levels of virginiamycin (0 and 22 mg/lamb).

The estimated final LW of the test subjects was calculated to be 41.50 kg, based on an ADG of 0.232 kg during the adaptation stage. We then determined the estimated average LW of the lambs, which was 37.52 kg throughout the 30-d experiment. This estimated ADG was used to calculate the daily dose of zilpaterol administered, with zilpaterol hydrochloride provided at a total dose of 7.55 mg/lamb daily for 28-d, followed by a three-d withdrawal period; and virginiamycin was supplemented for 30-d, followed by a one-d withdrawal period, which corresponded to the 24-hour fasting time prior to slaughter. Lambs were categorized by LW into four uniform weight groups (blocks) and allocated to 16 pens, with two lambs per pen, totaling eight lambs per treatment. LW measurements were taken two hours before the morning feeding during both the adaptation and experimental phases. A manual randomization method was employed by drawing ballots from containers to assign dietary treatments to pens within blocks, with four replicates per treatment. Each pen measured 6 m^2^ with overhead shade, 1 m fence-line feed bunk, and a manual waterer with *ad libitum* access.

### Experimental diets

Four treatments were randomly assigned to pens within each block: 1) control, no additives supplementation; 2) zilpaterol supplementation; 3) virginiamycin supplementation; and 4) zilpaterol plus virginiamycin supplementation. All lambs received an *ad libitum* diet (Table 1) based on cracked corn, providing 1.41 Mcal NEg/kg of DM and 14.1% of crude protein. Lambs were fed twice daily, at 09:00 and 15:00 h, with 30.0% of the total daily feed administered in the morning. A total daily dose of 7.55 mg/lamb of zilpaterol hydrochloride was administered by incorporating it into the morning feed for 28 d. In addition, 22 mg/lamb/d of virginiamycin was added to the feed both in the morning and afternoon throughout the 30-d experiment. Before each morning feeding, starting at 8:40, the uneaten feed from the previous day was visually evaluated to estimate the remaining quantity. If the amount of residual feed exceeded the established limit, the excess was removed and weighed. To limit feed waste, the quantity of feed provided to each pen was modified daily to maintain residual levels below 7.0%. Adjustments, either increases or decreases, were made during the afternoon feeding based on the observed intake.

### Calculations

Every day, feed samples were collected to analyze their DM content [20]. To evaluate the effects of treatments on growth performance, the LW of the lambs was recorded at the beginning and end of the experiment. To assess the impact of the treatments on carcass performance, the final LW-adjusted to HCW was calculated by dividing the HCW by the overall average dressing carcass (51.2%) across all treatments [21]. Results were expressed as ADG and FE, both adjusted to carcass final LW.

Considering that DM intake is related to energy requirements and the dietary NEm (Mcal/kg of DM), DM intake was estimated from ADG and LW values using the following equation: $\text{DM intake},\text{kg}/\text{d}=\left(\frac{\text{EM}}{2.07}\right)+\left(\frac{\text{EG}}{1.14}\right)$ Where EM (energy required for maintenance, Mcal/d) = EM = 56×SBW^0.75^, SBW, kg = (shrunk body weight, BW^0.96^), EG (energy required for gain, Mcal/d) = 276×ADG×SBW^0.75^ and NEm and NEg are 2.07 and 1.41 Mcal/kg of DM, respectively, derived from tabular values based on ingredient composition of the experimental diet [22]. A coefficient of 276 was estimated assuming a mature weight of 115 kg for Pelibuey×Katahdin male lambs [22]. Dietary net energy was estimated utilizing the quadratic formula [23]:


(1) $$x=\frac{-b-\sqrt{b^{2}-4ac}}{2c}$$

Where: x = NEm, a = −0.41 EM, b = 0.877 EM+0.41 DM intake+EG, and c = −0.877 DM intake.

### Carcass and organ mass

After completing the growth trial, the animals’ LW was recorded. The lambs were then transported in a small ruminant livestock trailer to the slaughterhouse facilities for carcass processing. Upon arrival, the animals were kept in a lairage pen with access to drinking water only. After slaughter, the HCW was recorded, and the dressing percentage was subsequently calculated. The carcasses were then stored in a cold room at 2°C for 24 h before being transported to the meat cutting room.

The carcasses were split longitudinally along the center of the vertebrae column. Subsequently, back fat thickness was measured using a digital vernier caliper (Cadena-a020) at a point located three-quarters of the length of the *longissimus dorsi* muscle from the split chine bone. In addition, the area of the *longissimus thoracis et lumborum* muscle (LTM) was determined using a plastic grid device placed on the cross-section of the left half of the carcass between the 12th and 13th ribs.

To determine the amount of perirenal and pelvic fat, it was manually removed from the carcass and expressed as a percentage of cold carcass weight. The left half of each carcass was then divided into primal cuts, and their proportions were calculated relative to the cold carcass weight. The primal cuts were obtained according to the guidelines of North American Meat Processors Association [24]. The forequarter was divided into the neck, foreshank (208D) shoulder (207), rib (209A), rack (204), and breast (209); while the hindquarter was divided into flank (232E), loin (232A), and leg (233A).

After processing the lambs and obtaining carcasses at the slaughterhouse, the organs and gastrointestinal components were separated into the stomach (rumen, reticulum, omasum, and abomasum), small intestine, large intestine, omental fat, mesenteric fat, and liver. The total digestive content was extracted and weighed to determine the weights of the full viscera, empty viscera, and the estimated empty body weight (EBW). The weight of each visceral component was expressed as a percentage of the EBW.

### Statistical analysis

Growth performance, dietary energetics, carcass characteristics, primal cuts, and visceral mass components data were analyzed using a randomized complete block design arranged as a 2×2 factorial experiment. The design included two levels of zilpaterol hydrochloride (0 and 0.20 mg/kg LW/d) and two levels of virginiamycin (0 and 22 mg/lamb/d), with initial LW as the blocking criterion. Each pen was considered the experimental unit. Data were analyzed using the MIXED procedure of the SAS software (SAS Institute).

The fixed effect was treatment, while pen was included as a random effect. Treatment effects were tested for: a) virginiamycin inclusion level (VIR) b) zilpaterol inclusion level (ZIL), and c) the virginiamycin×zilpaterol interaction (VIRxZIL). Additionally, to assess specific differences between treatments, the following contrasts were performed: VIR vs. ZIL, and VIR vs. VIRZIL. Statistical significance was declared at p≤0.05, and trends were considered when p≤0.10.

## RESULTS

The factorial analysis did not identify a significant interaction (VIR×ZIL) between virginiamycin and zilpaterol treatments in terms of growth performance, dietary energetics, carcass characteristics, or primal cut components. However, a significant interaction (p = 0.02) was observed for the empty large intestine weight within the visceral organ mass components

### Growth and dietary energetics

The main effects of virginiamycin and zilpaterol on growth performance are presented in Table 2. Virginiamycin supplementation showed significant improvements (p≤0.03) in growth performance, increasing final LW, total gain, ADG, and FE by 2.3%, 12.4%, 2.5%, and 9.6%, respectively, without affecting DM intake. Similarly, ADG and FE based on carcass-adjusted final LW improved with virginiamycin supplementation (p<0.01) by 16.8% and 13.5%, respectively.

Lambs treated with zilpaterol showed significant improvements (p<0.01) in final LW, total gain, ADG, and FE by 4.8%, 24.5%, 24.5%, and 20.0%, respectively, without affecting the DM intake. Additionally, ADG and FE adjusted to carcass-based final LW improved significantly (p<0.01) by 54.2% and 49.2%, respectively, compared to lambs not supplemented with zilpaterol.

The lambs that were supplemented with zilpaterol exhibited a 9.5% higher FE (p = 0.02) and showed a tendency (p = 0.06) to improve ADG by 10.8% compared to those receiving virginiamycin (VIR vs. ZIL). Similarly, ADG and FE adjusted to carcass-based final LW improved by 32.3% and 32.1%, respectively.

In a separate contrast (ZIL vs. VIRZIL), lambs that received the combination of virginiamycin plus zilpaterol exhibited greater (p≤0.03) ADG and FE than those supplemented with zilpaterol alone, with improvements of 12.8% and 9.4%, respectively. Moreover, ADG and FE, calculated from the final LW adjusted to carcass percentage increased by 32.3% and 32.1% respectively.

The effects of virginiamycin and zilpaterol treatments on dietary energetic components are presented in Table 3. The observed values of NEm and NEg in the diets of lambs supplemented with virginiamycin were significantly higher (p< 0.01) compared to those of lambs not receiving virginiamycin (5.7% and 6.9% respectively). Additionally, the observed-to-expected net energy ratio was greater by 5.6% for maintenance and 7.0% for gain.

Supplementation with zilpaterol improved the net energy observed in the diets of lambs compared to those not receiving the β-agonist, increasing NEm and NEg by 12.5% and 16.2%, respectively. Similarly, the observed-to-expected ratios for NEm and NEg increased by 12.7% and 16.0% respectively. Both additives, virginiamycin and zilpaterol, enhanced the efficiency of dietary energy utilization. However, zilpaterol supplementation increased the apparent NEm and NEg by 6.4% and 8.7%, respectively, compared to lambs treated with virginiamycin (VIR vs. ZIL). Furthermore, the simultaneous supplementation of virginiamycin and zilpaterol increased NEm and NEg by 5.6% and 6.7%, respectively, compared to lambs supplemented only with zilpaterol (ZIL vs. VIRZIL).

### Carcass and primal cuts

The effects of virginiamycin and zilpaterol supplementation on carcass characteristics are presented in Table 4. Virginiamycin supplementation resulted in a 3.1% increase in HCW compared to lambs not receiving virginiamycin (p<0.01), likely associated with improved FE. However, no significant effects were observed on dressing percentage, LTM area, or carcass fat traits.

Compared to untreated lambs, supplementation with zilpaterol significantly increased (p<0.01) HCW, dressing percentage, and LTM area by 9.4%, 4.5%, and 19.8%, respectively, while perirenal-pelvic fat decreased by 34.3%. These changes indicate improved muscle accretion and reduced fat deposition associated with zilpaterol supplementation.

Supplementation with zilpaterol increased (p<0.01) HCW by 6.2%, dressing percentage by 3.7%, and LTM by 20.3%, while perirenal-pelvic fat was reduced by 35.7% compared to lambs supplemented only with virginiamycin (VIR vs. ZIL). No differences in carcass fat thickness were observed when comparing virginiamycin and zilpaterol treatments. Lambs treated with both additives showed a 4.1% increase in HCW compared to those treated only with zilpaterol (ZIL vs. VIRZIL). No significant differences were observed in other carcass characteristics for this comparison.

The effects of virginiamycin and zilpaterol on primal cuts are presented in Table 5. Lambs treated with zilpaterol exhibited a reduction in the neck cut percentage (−13.1%; p = 0.01) and an increase in the leg cut percentage (3.6%; p = 0.04) compared to lambs not supplemented with zilpaterol. In contrast, virginiamycin supplementation did not affect any of the primal carcass cuts. Similarly, the neck cut was reduced (p = 0.02) in lambs treated with zilpaterol compared to those treated only with virginiamycin (VIR vs. ZIL), consistent with the main effect of zilpaterol. No significant differences were observed in primal cuts when comparing lambs supplemented with both additives to those supplemented only with zilpaterol (ZIL vs. VIRZIL).

### Visceral organ mass

The effects of virginiamycin and zilpaterol on EBW and visceral organ mass are presented in Table 6. Lambs supplemented with zilpaterol showed a 5.3% increase in EBW compared to lambs not receiving this additive (p = 0.01). In contrast, zilpaterol supplementation significantly reduced the relative weights of caul fat (p = 0.04), empty small intestine (p = 0.03), and liver (p = 0.02) by 24.7%, 16.8%, and 21.3%, respectively. Virginiamycin supplementation did not affect EBW or any visceral components. A significant interaction (p = 0.02) was observed between virginiamycin and zilpaterol in the percentage of the small intestine.

In the VIR vs. ZIL contrast, zilpaterol reduced the relative weights of empty full intestines, empty small intestine, caul fat, mesenteric fat, and liver by 15.9%, 17.5%, 31.8%, 45.3%, and 21.3%, respectively.

## DISCUSSION

### Growth performance and dietary energetics

The estimated zilpaterol hydrochloride dose was 0.20 mg/kg of LW per day, closely matching the actual intake calculated based on the average LW throughout the trial. The actual zilpaterol intake was 0.195 mg/kg LW for lambs receiving only zilpaterol and 0.192 mg/kg LW for those receiving both virginiamycin and zilpaterol.

The main effects indicate that supplementation with both virginiamycin and zilpaterol in fattening lambs improves growth performance, ADG, and FE without altering DM intake. No significant treatment interactions were observed for growth performance, dietary energetics, or carcass characteristics. However, lambs supplemented with both additives exhibited superior growth performance and energy utilization compared to those receiving either virginiamycin or zilpaterol alone. These outcomes are attributed to the complementary mechanisms of action of virginiamycin and zilpaterol: virginiamycin acts primarily by modulating ruminal fermentation, whereas zilpaterol enhances energy retention for muscle accretion.

Virginiamycin primarily enhances productivity by modulating the populations and activity of ruminal microorganisms, thereby promoting a stable fermentation pattern and maintaining DM intake in ruminants fed high-concentrate diets [9]. Additionally, virginiamycin effectively inhibits the growth of Gram-positive bacteria, particularly fast-growing lactic acid producers such as *Streptococcus bovis* and *Lactobacillus* spp., without affecting lactate-utilizing bacteria like *Megasphaera elsdenii* [10]. In addition to reducing lactic acid concentration in the rumen, virginiamycin supplementation positively alters the production of other volatile fatty acids, particularly increasing propionic acid levels in beef cattle [25]. Moreover, virginiamycin contributes to improved animal health by reducing the incidence of liver abscesses through the suppression of *Fusobacterium necrophorum* populations in the rumen. This bacterium, which can enter the portal circulation under ruminal acidosis conditions, becomes lodged in the liver and causes abscess formation [11]. Additionally, virginiamycin may prevent protein deamination in the rumen, thereby increasing the flow of undegraded protein to the small intestine and enhancing the supply of amino acids for tissue growth and muscle hypertrophy [26]. This improved amino acid availability, resulting from altered ruminal fermentation, could contribute to the enhanced growth and carcass performance observed with virginiamycin supplementation.

Recent studies have evaluated the use of virginiamycin supplementation in lambs, with an average supplementation of 25 mg per day, and have reported outcomes consistent with those of the present study [15,16]. Compared to control groups, virginiamycin supplementation improved FE by 7.3% to 13.8% and enhanced dietary energy utilization by up to 7.0%. However, only one study reported a significant increase in ADG of 27.0% following virginiamycin administration.

According to [2], the primary effect of zilpaterol on ruminant production is the improvement of both LW and dressing percentage. Zilpaterol activates a biological mechanism that enhances growth performance and energy retention by promoting muscle tissue deposition over adipose tissue. This effect is supported by the data presented in Table 3, which show an increase in dietary energy intake [22,23], as reflected in the apparent improvements in the observed-to-expected ratios for NEm, NEg, and DM intake. The enhancement of ADG and FE with zilpaterol supplementation has been extensively studied in lambs. A study [5] conducted on feedlot male lambs with an average LW of 34.98±0.24 kg, administered zilpaterol hydrochloride at a dose of 0.15 mg/kg of LW for 30 d, demonstrated increases in ADG and FE of 12.5% and 18.3%, respectively. Similarly, other authors [27] reported a 20.0% increase in ADG and a 26.9% improvement in FE in fattening male lambs averaging 29.3±0.21 kg supplemented with zilpaterol hydrochloride at a dose of 0.20 mg/kg of LW. These findings are consistent with the results of the present study, which yielded comparable productive outcomes.

### Carcass characteristics and primal cuts

Virginiamycin supplementation resulted in heavier HCW compared to lambs that did not receive this additive. However, virginiamycin had no significant effect on dressing percentage, LTM area, fat thickness, renal-pelvic fat, or primal cut yields. It may be hypothesized that virginiamycin could have a greater impact on carcass quality if administered over longer periods and during early growth stages, when excess dietary protein has greater biological value for promoting tissue accretion. Research on virginiamycin use in feedlot lambs remains limited. In a study by [15], no significant effects were observed on HCW, dressing percentage, or LTM area when finishing lambs were supplemented with 25 mg/lamb of virginiamycin for 87 d. Similarly, studies in cattle suggest that virginiamycin supplementation does not consistently improve carcass quality traits [28,29]. However, one cattle experiment [30] using 22.5 mg/kg of virginiamycin reported improvements in HCW, dressing percentage, and LTM, although such results remain inconsistent.

The biological effects of β-adrenergic agonists occur in peripheral tissues or cells that express β-adrenergic receptors. Most of their actions are mediated by increased intracellular cyclic adenosine monophosphate, as the binding of a β-adrenergic agonists to its receptor activates adenylate cyclase through the stimulatory Gs protein pathway. Zilpaterol, a β-agonist, exerts its effect through high affinity for β_2_-adrenergic receptors located on skeletal muscle cells and adipocytes. It reduces adipose tissue by inhibiting fatty acid synthesis and stimulating lipolysis through the breakdown of triacylglycerols. Additionally, zilpaterol promotes muscle hypertrophy by enhancing protein synthesis and reducing proteolysis [2]. As expected, zilpaterol supplementation in the present study enhanced muscle development and reduced adipose tissue in lamb carcasses. These findings are consistent with previous reports [4,31] in which zilpaterol hydrochloride supplementation (0.20 mg/kg of LW) in feedlot male lambs (approximately 37.5 kg of LW) increased HCW by 4.6% to 6.1%, dressing percentage by up to 2.6%, and LTM by 1.7% to 13.7%.

Beta-agonist supplementation enable meat-producing animals to synthesize muscle protein beyond their genetic potential. This has been demonstrated in previous lamb studies [5,21], where zilpaterol supplementation increased LW and HCW by up to 7.0% and 4.8%, respectively. The present findings are consistent with earlier research showing a 9.4% improvement in LW and a 4.5% increase in HCW. The energy redistribution between adipose and muscle tissues was also evident in the increased LTM area (19.9%) and reduced perirenal-pelvic fat (−35.0%), observed in this study. These results are in line with those reported by [32] who documented a 33.9% increase in LTM and reduction in perirenal-pelvis fat.

In our experiment, zilpaterol supplementation in lambs resulted in a 4.0% increase in the leg primal cut. Although zilpaterol is known to enhance HCW and dressing percentage, significant changes in individual primal cuts are not consistently reported [5,33]. This inconsistency may be due to the fact that the muscle hypertrophy induced by zilpaterol is distributed across the carcass, producing differential effects in specific muscle regions. Some studies suggest that zilpaterol may enhance hindquarter development in lambs. According to [27], dietary inclusion of 0.20 mg/kg of LW/d of zilpaterol hydrochloride increased the leg primal cut by 2.3% compared to the control group. Additionally, an average intake of 0.29 mg/kg of LW/d of zilpaterol improved the same cut by 1.5%. Another study by [27] reported that an average intake of 0.15 mg/kg of LW/d of zilpaterol hydrochloride resulted in a 12.6% increase in hindquarter weight.

### Visceral organ mass

A consistent response to zilpaterol supplementation on non-carcass components is a reduction in liver weight among treated animals. Several authors have reported a reduction in liver weight ranging from 6.1% to 19.2% in lambs treated with different doses of zilpaterol [21,34,35]. Although extensive information exists regarding the use of zilpaterol in ruminants, a reduction in proportion in the small intestine in lambs or cattle treated with zilpaterol rarely been reported [5,21,35]. Nevertheless, one study [27] documented a 16.7% decrease in the weight of the empty small intestine, which is consistent with the finding of the present study.

**Figure 1:**
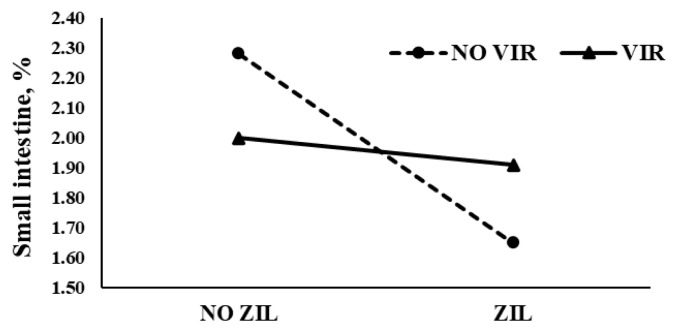
Interaction effect of zilpaterol and virginiamycin on small intestine percentage. ZIL, zilpaterol hydrochloride (0.20 mg/kg LW) 28 d of supplementation plus 3 d of withdrawal; VIR, virginiamycin (22 mg/lamb) 30 d of supplementation plus 1 d of withdrawal.

Zilpaterol is classified as an energy repartitioning agent, primarily recognized for its capacity to reduce overall body fat. Studies in lambs and cattle have consistently reported reductions in visceral and perirenal fat [18,21]. Therefore, the observed decrease in small intestine weight may be attributed to a reduction in mesenteric fat induced by zilpaterol administration. Although a portion of the mesenteric fat is manually removed during the weighing process, complete removal is not feasible, as the remaining fat is also subject to zilpaterol’s effect and may influence its relative proportion. Figure 1 illustrates the interaction effect between zilpaterol and virginiamycin on the relative weight of the small intestine. This result is particularly relevant because, unlike the consistent reduction in visceral fat typically attributed to zilpaterol, the combination with virginiamycin appears to attenuate this effect. Given that virginiamycin enhances dietary energy availability, the absence of a reduction in small intestine weight may reflect an increased mesenteric fat deposition surrounding this organ. Fat accumulation in visceral tissues is closely related to the availability of surplus energy, and based on the observed net energy values (NEm and NEg), virginiamycin supplementation appears to promote greater energy intake, potentially overriding the repartitioning effect of zilpaterol [9,26]. Although zilpaterol has been shown to reduce overall body fat, including carcass and visceral depots, its effectiveness is known to depend on factors such as dosage and duration of administration. In the present study, it is reasonable to assume that the dose and exposure period of zilpaterol were insufficient to counteract the elevated energy availability induced by virginiamycin, resulting in maintained or increased small intestine weight.

## CONCLUSION

Zilpaterol hydrochloride supplementation at a dose of 0.20 mg/ of LW/d, equivalent to 7.55 mg/lamb/d enhances muscle development, as reflected in improved growth performance, dietary energetics, and carcass quality traits. Additionally, its lipolytic effect contributes to the reduction of body fat, including pelvic-renal fat, caul fat, and the relative proportion of the small intestine. Virginiamycin supplementation at 22 mg/lamb/d improves growth performance and dietary energy retention without affecting carcass quality traits, primal cut yields, or non-carcass components.

Since zilpaterol and virginiamycin exert their effects through different physiological mechanisms, their combined use resulted in increases of 5.6% and 6.7% in apparent NEm and NEg, respectively. This combination improved growth performance and FE without altering DM intake, which may contribute to reduced feed costs and increased profitability in lamb production systems. By promoting muscle accretion over fat deposition, this strategy enhances lean meat yield and dressing percentage, thereby enhancing the value of lamb meat quality for final processing. These findings suggest that future research should evaluate different doses of zilpaterol supplementation in combination with virginiamycin, considering the increased energy availability resulting from virginiamycin’s effect. Additional areas of interest for the combined use of zilpaterol and virginiamycin include assessing the duration of virginiamycin administration and the potential benefits of initiating supplementation during early growth stages.

## Figures and Tables

**Table 1:** Ingredient and chemical composition of experimental diet

Ingredient composition (%, DM basis)	Control	VIR	ZIL	VIR×ZIL
Cracked corn	69.5	69.5	69.5	69.5
Sudangrass hay	12.0	12.0	12.0	12.0
Soybean meal	6.7	6.7	6.7	6.7
Molasses cane	5.0	5.0	5.0	5.0
Urea	0.8	0.8	0.8	0.8
Tallow	3.5	3.5	3.5	3.5
Trace mineral salt	2.5	2.5	2.5	2.5
Virginiamycin		*		*
Zilpaterol			*	*
Nutritional composition^1)^				
Dry matter	88.2	88.2	88.2	88.2
Neutral detergent fiber	16.4	16.4	16.4	16.4
Crude protein	14.1	14.1	14.1	14.1
Ether extract	6.7	6.7	6.7	6.7
Calculated net energy (Mcal/kg)				
Maintenance	2.07	2.07	2.07	2.07
Gain	1.41	1.41	1.41	1.41

1) Derived from tabular values based on ingredient composition of the experimental diet.

* Additives administered daily with the feed: Zilpaterol hydrochloride, 0.20 mg/kg LW; Virginiamycin, 22 mg/lamb.

DM, dry matter; VIR, virginiamycin inclusion level; ZIL, zilpaterol inclusion level.

**Table 2:** Treatments effects of virginiamycin and zilpaterol on growth performance on feedlot lambs

	Treatments^1)^					p-value				

	NO ZIL		ZIL		SEM	Main effects			Contrasts	
			
	NO VIR	VIR	NO VIR	VIR		VIR	ZIL	VIR×ZIL	VIR vs. ZIL	ZIL vs. VIRZIL
Initial LW	34.00	33.96	34.12	34.07	1.05	0.82	0.57	1.00	0.57	0.87
Final LW	41.38	42.21	43.26	44.39	1.12	0.03	<0.01	0.72	0.10	0.08
Total gain (kg)	7.37	8.25	9.14	10.31	0.32	<0.01	<0.01	0.64	0.06	0.02
ADG (kg)	0.264	0.290	0.327	0.369	0.01	<0.01	<0.01	0.62	0.06	0.02
DMI (kg/d)	1.20	1.21	1.22	1.27	0.02	0.34	0.23	0.67	0.84	0.34
DMI (% of LW)	3.19	3.21	3.19	3.25	0.05	0.53	0.72	0.72	0.84	0.49
FE	0.221	0.243	0.266	0.291	0.01	<0.01	<0.01	0.30	0.02	0.03
Adjust carcass^2)^										
Final LW	40.51	41.31	43.87	45.71	1.25	0.01	<0.01	0.38	<0.01	0.01
ADG	0.233	0.263	0.348	0.416	0.02	<0.01	<0.01	0.36	<0.01	<0.01
FE	0.195	0.215	0.284	0.329	0.01	0.01	<0.01	0.42	<0.01	0.08

1) ZIL, zilpaterol hydrochloride (0.20 mg/kg LW) 28 d of supplementation plus 3 d of withdrawal; VIR, virginiamycin (22 mg/lamb) 30 d of supplementation plus 1 d of withdrawal.

2) Growth performance components calculated from final LW-adjusted carcass.

LW, live weight; ADG, average daily gain; DMI, dry matter intake; FE, gain/feed efficiency.

**Table 3:** Treatments effects of virginiamycin and zilpaterol on dietary energetics calculation on feedlot lambs

	Treatments^1)^					p-value				
	
	NO ZIL		ZIL		SEM	Main effects			Contrasts	
			
	NO VIR	VIR	NO VIR	VIR		VIR	ZIL	VIR×ZIL	VIR vs. ZIL	ZIL vs. VIRZIL
NEm observed^2)^	2.06	2.18	2.32	2.45	0.04	<0.01	<0.01	0.89	0.02	0.03
NEg observed	1.40	1.50	1.63	1.74	0.03	<0.01	<0.01	0.89	0.02	0.03
Ob/ex DMI^3)^	1.00	0.93	0.87	0.82	0.02	<0.01	<0.01	0.69	0.01	0.03
Ob/ex NEm ratio^4)^	0.99	1.05	1.12	1.18	0.02	<0.01	<0.01	0.90	0.02	0.03
Ob/ex NEg ratio	0.99	1.06	1.15	1.23	0.02	<0.01	<0.01	0.89	0.02	0.03

1) ZIL, zilpaterol hydrochloride (0.20 mg/kg LW) 28 d of supplementation plus 3 d of withdrawal; VIR, virginiamycin (22 mg/lamb) 30 d of supplementation plus 1 d of withdrawal.

2) NE, net energy of maintenance and gain (Mcal/kg DM) observed.

3) Observed/expected (calculated) ratio of DM intake.

4) Observed/expected ratio of net energy.

**Table 4:** Treatments effects of virginiamycin and zilpaterol on carcass characteristics on feedlot lambs

	Treatments^1)^					p-value				

	NO ZIL		ZIL		SEM	Main effects			Contrasts	
			
	NO VIR	VIR	NO VIR	VIR		VIR	ZIL	VIR×ZIL	VIR vs. ZIL	ZIL vs. VIRZIL
Hot carcass weight (kg)	20.76	21.17	22.48	23.42	0.64	<0.01	<0.01	0.22	<0.01	0.01
Dressing (%)	50.10	50.10	51.94	52.81	0.36	0.27	<0.01	0.28	<0.01	0.13
LTM area (cm^2^)	25.78	25.81	31.05	30.78	1.03	0.89	<0.01	0.86	<0.01	0.83
Fat thickness (cm)	2.64	2.19	2.37	2.74	0.15	0.90	0.69	0.27	0.71	0.47
Perirenal-pelvis fat (%)	2.58	2.49	1.60	1.73	0.12	0.85	<0.01	0.39	<0.01	0.46

1) ZIL, zilpaterol hydrochloride (0.20 mg/kg LW) 28 d of supplementation plus 3 d of withdrawal; VIR, virginiamycin (22 mg/lamb) 30 d of supplementation plus 1 d of withdrawal.

LTM, *longissimus thoracis et lumborum*.

**Table 5:** Treatments effects of virginiamycin and zilpaterol on primal cuts as percentage of HCW on feedlot lambs

Primal cuts^2)^	Treatments^1)^					p-value				
	
	NO ZIL		ZIL		SEM	Main effects			Contrasts	
			
	NO VIR	VIR	NO VIR	VIR		VIR	ZIL	VIR×ZIL	VIR vs. ZIL	ZIL vs. VIRZIL
Forequarter	53.59	53.37	51.92	52.67	0.30	0.68	0.09	0.46	0.13	0.42
Hindquarter	46.41	46.63	48.08	47.33	0.30	0.68	0.09	0.46	0.13	0.42
Neck^3)^	5.18	5.12	4.51	4.44	0.11	0.69	<0.01	0.96	0.02	0.76
Foreshank-208D	19.43	19.33	19.62	19.23	0.18	0.76	0.88	0.75	0.64	0.54
Rib-209A	6.76	6.58	6.54	6.64	0.10	0.86	0.77	0.60	0.92	0.79
Rack-204	8.43	8.29	8.10	8.33	0.09	0.84	0.49	0.36	0.51	0.42
Breast-209	3.50	3.50	3.76	3.37	0.14	0.50	0.81	0.51	0.53	0.36
Shoulder-207	10.26	10.52	9.38	10.59	0.23	0.12	0.34	0.33	0.11	0.10
Flank-232E	6.80	7.35	6.74	6.93	0.13	0.20	0.40	0.52	0.13	0.61
Loin-232-A	8.96	8.48	9.27	8.81	0.14	0.16	0.32	0.98	0.10	0.32
Leg-233A	30.65	30.81	32.05	31.63	0.26	0.62	0.04	0.60	0.12	0.58

1) ZIL, zilpaterol hydrochloride (0.20 mg/kg LW) 28 d of supplementation plus 3 d of withdrawal; VIR, virginiamycin (22 mg/lamb) 30 d of supplementation plus 1 d of withdrawal.

2) NAMP, North American Meat Processors Association number item.

3) Not included in NAMP’s list as a primal cut.

HCW, hot carcass weight.

**Table 6:** Treatments effects of virginiamycin and zilpaterol on visceral organ mass on feedlot lambs

	Treatments^1)^					p-value				

	NO ZIL		ZIL		SEM	Main effects			Contrasts	
			
	NO VIR	VIR	NO VIR	VIR		VIR	ZIL	VIR×ZIL	VIR vs.ZIL	ZIL vs. VIRZIL
EBW (kg)	37.27	38.09	39.54	39.85	0.64	0.40	0.01	0.70	0.14	0.74
As % of EBW										
Empty body	90.24	90.12	91.38	89.57	1.03	0.37	0.77	0.43	0.40	0.24
Digestive content	9.75	9.88	8.61	10.42	1.03	0.37	0.77	0.43	0.40	0.24
Full rumen	12.60	12.72	10.70	13.46	1.25	0.28	0.65	0.31	0.28	0.15
Empty rumen	4.40	3.86	3.69	4.24	0.33	0.99	0.63	0.14	0.72	0.28
Full intestines	7.30	6.61	6.22	6.85	0.42	0.94	0.34	0.15	0.53	0.32
Empty LI	2.34	2.45	2.10	2.46	0.15	0.16	0.47	0.44	0.14	0.13
Empty SI	2.28	2.00	1.65	1.91	0.10	0.90	<0.01	0.02	0.04	0.11
Caul fat	1.70	1.57	1.07	1.39	0.14	0.51	0.01	0.15	0.03	0.15
Mesenteric fat	0.42	0.53	0.29	0.43	0.08	0.15	0.20	0.87	0.06	0.25
Liver	4.58	4.45	3.50	3.60	0.17	0.96	<0.01	0.60	0.01	0.75

1) ZIL, zilpaterol hydrochloride (0.20 mg/kg LW) 28 d of supplementation plus 3 d of withdrawal; VIR, virginiamycin (22 mg/lamb) 30 d of supplementation plus 1 d of withdrawal.

EBW, empty body weight; LI, large intestine; SI, small intestine.
